# β-Adrenergic Receptor Stimulation Maintains NCX-CaMKII Axis and Prevents Overactivation of *IL6R*-Signaling in Cardiomyocytes upon Increased Workload

**DOI:** 10.3390/biomedicines10071648

**Published:** 2022-07-08

**Authors:** Ingrid Matzer, Julia Voglhuber, Mara Kiessling, Nataša Djalinac, Viktoria Trummer-Herbst, Nishani Mabotuwana, Lavinia Rech, Michael Holzer, Samuel Sossalla, Peter P. Rainer, Andreas Zirlik, Senka Ljubojevic-Holzer

**Affiliations:** 1 Department of Cardiology, Medical University of Graz, 8036 Graz, Austria; ingrid.matzer@medunigraz.at (I.M.); mara.kiessling@medunigraz.at (M.K.); natasa.djalinac@medunigraz.at (N.D.); viktoria.herbst@medunigraz.at (V.T.-H.); nishani.mabotuwana@stud.medunigraz.at (N.M.); cara.rech@medunigraz.at (L.R.); peter.rainer@medunigraz.at (P.P.R.); andreas.zirlik@medunigraz.at (A.Z.); 2 BioTechMed-Graz, 8010 Graz, Austria; michael.holzer@medunigraz.at; 3 College of Health, Medicine and Wellbeing, The University of Newcastle, Newcastle, NSW 2308, Australia; 4 Hunter Medical Research Institute, Newcastle, NSW 2305, Australia; 5 Otto-Loewi Research Centre, Division of Pharmacology, Medical University of Graz, 8036 Graz, Austria; 6 Department of Internal Medicine II, University Medical Centre Regensburg, 93053 Regensburg, Germany; samuel.sossalla@klinik.uni-regensburg.de; 7 Gottfried Schatz Research Center, Division of Molecular Biology and Biochemistry, Medical University of Graz, 8010 Graz, Austria

**Keywords:** β-adrenergic signaling, isoprenaline, CaMKII, *IL6R*, cardiomyocyte, hypertrophy, hypertensive cardiomyopathy

## Abstract

Excessive β-adrenergic stimulation and tachycardia are potent triggers of cardiac remodeling; however, their exact cellular effects remain elusive. Here, we sought to determine the potency of β-adrenergic stimulation and tachycardia to modulate gene expression profiles of cardiomyocytes. Using *neonatal rat ventricular cardiomyocytes*, we showed that tachycardia caused a significant upregulation of sodium–calcium exchanger (NCX) and the activation of calcium/calmodulin-dependent kinase II (CaMKII) in the nuclear region. Acute isoprenaline treatment ameliorated NCX-upregulation and potentiated CaMKII activity, specifically on the sarcoplasmic reticulum and the nuclear envelope, while preincubation with the β-blocker propranolol abolished both isoprenaline-mediated effects. On a transcriptional level, screening for hypertrophy-related genes revealed tachycardia-induced upregulation of interleukin-6 receptor (*IL6R*). While isoprenaline prevented this effect, pharmacological intervention with propranolol or NCX inhibitor ORM-10962 demonstrated that simultaneous CaMKII activation on the subcellular Ca^2+^ stores and prevention of NCX upregulation are needed for keeping *IL6R* activation low. Finally, using hypertensive Dahl salt-sensitive rats, we showed that blunted β-adrenergic signaling is associated with NCX upregulation and enhanced *IL6R* signaling. We therefore propose a previously unrecognized protective role of β-adrenergic signaling, which is compromised in cardiac pathologies, in preventing *IL6R* overactivation under increased workload. A better understanding of these processes may contribute to refinement of therapeutic options for patients receiving β-blockers.

## 1. Introduction

Cardiac remodeling encompasses changes at the molecular, cellular and transcriptional level following pathologic insult to the heart and is characterized by gradual functional and structural decline [1]. Excessive β-adrenergic stimulation and tachycardia are potent triggers of cardiac remodeling [2,3,4,5], and while many aspects of their cellular effects are well understood, others remain elusive. Initially, elevated β-adrenergic stimulation signals the heart to use its chronotropic and inotropic reserve, thereby stabilizing blood pressure and cardiac output and allowing patients to remain asymptomatic for a limited post-insult time [2]. If prolonged, however, it leads to progressive decline in contractile function, constituting a vicious cycle that eventually causes organ failure.

β-adrenergic stimulation and tachycardia are well documented to increase intracellular calcium (Ca^2+^) cycling in cardiomyocytes [4,6]. Although this is critical for enhancing myofilament contraction and matching the increased energy demands by stimulating mitochondrial ATP production, little is known about the effects of β-adrenergic stimulation-mediated increase in intracellular Ca^2+^ on transcriptional regulation in the process of excitation–transcription coupling [7,8]. Excitation–transcription coupling is a very early key feature of cardiac remodeling in cardiomyocytes [4,9,10], and it conveys the activation of ion-handling molecules at the sarcolemma into transcriptional reprograming with subsequent structural and functional consequences [5,11,12]. Several Ca^2+^-handling molecules have been implicated in excitation–transcription coupling and the subsequent development of hypertrophy. In our previous work, we systematically studied the contribution of sodium–calcium exchanger (NCX), sodium–proton exchanger (NHE), sodium channel (Nav 1.5), sodium potassium pump (Na^+^/K^+^ ATPase), L-type calcium channel (LTCC), transient receptor potential channel (TRP), calcium/calmodulin-dependent protein kinase II (CaMKII) and calcineurin to hypertrophic remodeling due to tachycardia [5]. We demonstrated that NCX most prominently contributed to tachycardia-mediated alterations in Ca^2+^-mediated transcriptional regulation and that this effect was linked to CaMKII activation via phosphorylation that occurred downstream of NCX. Despite the correlation of tachycardia with increased β-adrenergic tone and β-blocker therapy being a mainstay of the pharmacological treatment of heart failure [2], the contribution of β-adrenergic signaling to the processes of excitation–transcription coupling under the conditions of increased workload and tachycardia has so far not been comprehensively documented.

Here, we aimed to study the individual and synergistic potency of β-adrenergic stimulation and tachycardia to modulate pathological gene expression profiles, as well as the efficacy of β-blocker treatment in preventing these alterations. Using *neonatal rat ventricular cardiomyocytes (NRVCMs)*, we showed that β-adrenergic signaling can—via limiting NCX upregulation and potentiating CaMKII activity specifically on the sarcoplasmic reticulum and the nuclear envelope—prevent the upregulation of interleukin-6 receptor (*IL6R*) under increased workload. Furthermore, we showed that blunted β-adrenergic signaling is associated with NCX upregulation and enhanced *IL6R* signaling in hypertensive Dahl salt-sensitive rats. We propose a previously unrecognized protective role of β-adrenergic signaling in preventing *IL6R* upregulation and NCX dysregulation under increased workload, molecular mechanisms that are severely disrupted in cardiac pathologies. A better understanding of these processes may contribute to refinement of patient care, with special regard to the use of β-blockers.

## 2. Materials and Methods

### 2.1. Ethical Consideration

All procedures involving animals conformed to the EU Directive 2010/63/EU and were approved by the institutional ethical committee and the Austrian Federal Ministry of Education, Science and Research (Vienna, Austria; BMBWF-66.010/0147-V/3b/2018).

### 2.2. Isolation and Culture of Neonatal Rat Ventricular Cardiomyocytes

Isolation and culture of *neonatal rat ventricular cardiomyocytes (NRVCMs)* were performed as previously described [5,9], with minor adaptations. Briefly, female rats with timed pregnancy were purchased from Charles River Laboratories (Sulzfeld, Germany), and hearts were excised from 1-day-old Wistar pups (15-17 pups per isolation) following cervical dislocation. Hearts were then minced and digested using a liberase-based approach (digestion solution: 1X HBSS, 0.08% Liberase^TM^ Research Grade (Merck, Darmstadt, Germany), 0.1% Trypsin Gibco^TM^ (Thermo Fisher Scientific, Waltham, MA, USA), 0.2% DNAse I (Merck, Darmstadt, Germany), 10 µM CaCl_2_ and 20 mM HEPES; pH 7.3) at 37 °C and 0% CO_2_. After 10 min of digestion, the supernatant was discarded, and fresh digestion solution was added to the remaining pieces four times for 15 min. The supernatant was collected and digestion was stopped by adding 10% horse serum Gibco^TM^ (Thermo Fisher Scientific, Waltham, MA, USA). Isolated cells were centrifuged in a Heraeus multifuge (Thermo Fisher Scientific, Waltham, MA, USA) for 8 min at 350× *g*, and pellets were resuspended in culture medium (DMEM/F12, GlutaMAX^TM^ containing 10% FBS and 1% Penicillin/Streptomycin) and finally pooled for further processing.

After filtering through a 100 µm nylon mesh filter, cells were pre-plated in cell culture flasks (90 min at 37 °C and 5% CO_2_) to remove potential non-myocytes. NRVCMs were plated at a density of 10^6^ cells/ml on gelatin-plated 35 mm glass-bottom dishes for immunocytochemistry (Greiner Bio-One, Kremsmünster, Austria) or plastic-bottom dishes for qPCR (Greiner Bio-One, Kremsmünster, Austria). One day post-isolation, media was exchanged for serum-free media (DMEM/F12, GlutaMAX^TM^ containing 20% Medium M199/GlutaMAX^TM^ and Penicillin/Streptomycin). NRVCMs were ultimately cultured for 3 days prior to induction of acute experimental conditions.

NRVCMs were electrically stimulated with carbon electrodes (C-pace EP, IonOptix, Westwood, KS, USA) for 3 h at 37 °C and 5% CO_2_ at either 1 Hz (CTL) or 8 Hz (Tachycardia, TC) in control media or media containing 10 µM isoprenaline (ISO), 10 µM ISO after 1 h preincubation with 1 µM propranolol (β-blocker, ISO + BB) or 1 µM ORM-10962 (NCX inhibitor, ORM). Cell contractility was monitored using a conventional light microscope.

### 2.3. Rat Model of Hypertension, In Vivo Characterization Methods and Gravimetry

Male Dahl Salt-Sensitive (DSS) rats were purchased from Charles River Laboratory (Boston, MA, USA) at 4–5 weeks of age. Starting at 7 weeks of age, rats were fed either a high-salt diet (HSD, 8% NaCl; Research Diets; D05032408Y) to induce hypertension or a low-salt diet (LSD, 0.3% NaCl; Research Diets; D10001R) as a control (see Figure S1A in the Supplementary Materials for a schematic overview of the feeding and experimental protocol). Rats remained on their respective diets for 10 weeks to study hypertension-induced cardiac remodeling.

Blood pressure (BP) measurements were carried out biweekly using the CODA^®^ tail cuff system (Kent Scientific Corporation, Torrington, CT, USA). Recordings were performed in steady-state conditions, and values from three consecutive measurements were averaged.

Cardiac function was assessed via transthoracic echocardiography [10] using Vevo 3100 (VisualSonics, Toronto, ON, Canada) with rats under light anesthesia (Isoflurane, 1-1.5% and 1.5–2 l/min oxygen flow). For determination of ejection fraction (EF) and left ventricular (LV) mass in M-mode, images were acquired in the parasternal long axis. To assess diastolic function, the ratio of peak early Doppler transmitral flow velocity (E) to myocardial-tissue Doppler velocity (e′), images using pulsed-wave Doppler and tissue Doppler mode were acquired in a modified parasternal long axis and parasternal short axis, respectively.

After euthanasia by cervical dislocation of heavily anesthetized rats, organs were collected and gravimetric measurements of body weight (BW) and heart weight (HW) were noted.

### 2.4. Isolation of Adult Cardiomyocytes

For isolation of ventricular cardiomyocytes from adult Dahl salt-sensitive rats, a liberase-based Langendorff perfusion protocol as previously described for mice [4,6] was optimized and modified.

In detail, the thoracic cavity of heparinized animals (500IE) was opened after sacrifice, and the heart was rapidly excised and submerged in ice-cold perfusion buffer (PB, in mM: 135 NaCl, 4.7 KCl, 0.6 KH_2_PO_4_, 0.6 HNa_2_PO_4_dibasic, 1.2 MgSO_4_*7H_2_O, 10 HEPES, 30 Taurin, 10 BDM, 10 Glucose) supplemented with 1 mM CaCl_2_. The aorta was cannulated for retrograde perfusion and the coronaries flushed of remaining blood. Following 1.5 min of perfusion with temperature-controlled Ca^2+^-free solution (PB) at 37 °C, hearts were digested using 6 mg/ml Liberase^TM^ Research Grade (Merck, Darmstadt, Germany) and 0.014% Trypsin Gibco^TM^ (Thermo Fisher Scientific, Waltham, MA, USA) in PB with 12.5 µM CaCl_2_ for 9–12 min (depending on visible signs of digestion). Digestion was stopped by submerging ventricles in a bovine calf serum (BCS)-containing stop solution (PB with 12.5 µM CaCl_2_ and 10% BCS), and the tissue was carefully dissociated using a cut and blunted Pasteur pipette and sieved through a 300 µm mesh. Cardiomyocytes were allowed to settle by gravity before exposure to a series of increasing [Ca^2+^] (PB with 5.0% BCS, and CaCl_2_ in 5 steps), reaching a maximum of 1.5 mM CaCl_2_. For subsequent immunocytochemistry [10], cells were plated on laminin-coated glass-bottom dishes (prepared the night before) and allowed to attach for 1 h at room temperature in the final CaCl_2_ solution before exposure to Normal Tyrode’s solution (NT, in mM: 140 NaCl, 4 KCl, 1 MgCl_2_*6H_2_O, 10 HEPES, 1.5 CaCl_2_, 5 Glucose) at 37 °C and 5% CO_2_ for a subset of cells pacing at 0.2 Hz for 1 h (C-pace EP, IonOptix, Westwood, KS, USA).

### 2.5. Immunocytochemistry and Confocal Imaging

To fix NRVCMs, cell culture media was rapidly removed, and cells were exposed to 4% PFA/PBS for 10 min. For adult cardiomyocytes, concentrated PFA was added directly to NT, and cells were fixed in a solution ultimately containing 1% PFA for 10 min.

Permeabilization was performed in 0.1% (for NRVCMs) or 0.6% (for adult cardiomyocytes) Triton X-100 in PBS for 10 min at RT, and cells were subsequently washed in PBS. Incubation with primary antibody (anti-NCX1, 1:300, R3F1, Swant, Burgdorf, Switzerland; anti-pCaMKIIT286, 1:500, ab32678, or anti-ADRB1, 1:100, ab3442, both Abcam, Cambridge, UK) in a solution containing 5% BSA, 3% goat serum and 0.01% Triton X-100 in PBS was performed for either 4–5 h at RT or overnight at 4 °C. Cells were then washed twice in 0.01% Triton X-100 in PBS and incubated with secondary fluorescent antibody (anti-rabbit Alexa647, 1:1000, cs4414; anti-mouse Alexa488, 1:500, cs4408, both Cell Signaling Technology, Danvers, MA, USA, or anti-rabbit Alexa647, 1:500, ab150075, Abcam, Cambridge, UK) for 1.5 h at RT and protected from light. Cells were washed twice in 0.01% Triton X-100 and twice in PBS before staining with DAPI (1:200) for 10 min at RT. Finally, cells were washed 3 times in PBS and imaged using a confocal fluorescence microscope (LSM 510 Meta with a Plan Neofluar 40x/1.3 oil-immersion objective, Carl Zeiss, Jena, Germany). Image J software (NIH, Bethesda, MD, USA) was used to measure mean fluorescence intensity in the region of interest. To minimize effects of cohort variability and antibody inter-batch variability, background-subtracted signals were normalized to mean of the corresponding controls. Hypertrophy measures were taken by measuring cell length and width with the measuring tool in Image J.

### 2.6. Isolation of RNA and Quantitative Real-Time PCR (qPCR) of NRVCMs

Cells were scraped from plastic-bottom dishes and lysed in Qiazol^TM^ lysis reagent, and total RNA was isolated with the miRNeasy^®^ Kit (Qiagen, Hilden, Germany). RNA yield and quality were assessed with the NanoDrop^TM^ 2000 (Thermo Fisher Scientific, Waltham, MA, USA). Subsequent gDNA wipeout and cDNA synthesis using the QuantiTect Reverse Transcription Kit (Qiagen, Hilden, Germany) were carried out in a peqStar Thermocycler (VWR, Radnor, PA, USA). cDNA was diluted to a working concentration of 10 ng per reaction volume for qPCR. qPCR was performed using the TaqMan-System (TaqMan^TM^ Gene Expression Master Mix and TaqMan^TM^ Assays; Thermo Fisher Scientific, Waltham, MA, USA; primers listed in Table S1) according to the manufacturer’s instructions, and a Bio-Rad CFX384 Touch Real-Time PCR Detection System (Bio-Rad Laboratories Inc., Hercules, CA, USA). GenEx 5.4.4. (MultiD, Gothenburg, Sweden) was used for efficiency correction on all primer sets, followed by relative quantification of gene expression with the 2^−ΔΔCt^ quantification method. GAPDH served as the housekeeping gene. All procedures were carried out according to the MIQE guidelines [13].

### 2.7. Protein Extraction and Immunoblotting of Cardiac Tissue from Adult Dahl Salt-Sensitive Rats

Tissue samples were homogenized utilizing freshly prepared ice-cold homogenization buffer (HP in mM: 137 NaCl, 20 Tris-HCl pH 7.4, 20 NaF, 1 Na-orthovandat, 1 Na-pyrophsophat, 50 β-glycerolphosphat, 10 EDTA pH 8, 1 EGTA pH 7, 1 PMSF; 1% IGEPAL, 10% glycerol, 4 µg/mL each: aprotinin, leupeptin and pepstatin A) by mincing and the use of an electric homogenizer. After collecting the supernatant in two short centrifugation steps at 8000 rpm at 4 °C, the homogenate was centrifuged at full speed for 10 min at 4 °C to collect the protein fraction in the supernatant. Protein concentration was determined by Pierce^TM^ BCA Protein Assay Kit (ThermoFisher Scientific, Waltham, MA, USA) according to the manufacturer’s protocol, and the absorbance was measured at 562 nm with a SpectraMax^®^ Plus 374 photometer (Avantor, VWR, Radnor, PA, USA).

For immunoblotting, 30 µg of sample underwent electrophoresis using a Bis-Tris/MOPS-based approach and 4–12% gradient gels. Electrophoresis was initiated at 70 V for 10 min and then increased to 120 V for approximately 2.5 h on ice. Protein transfer was carried out for 2 h at 400 mA and 4 °C using ice-cold transfer buffer (in mM: 192 glycine, 25 Tris, 20% Methanol). Total protein content was detected by staining membranes for 3–5 min in Ponceau S and documented using the ChemiDoc^TM^ Touch Imaging System (Bio-Rad Laboratories Inc, Hercules, CA, USA). Blocking was performed with 5% milk powder in TBST for 1 h at RT before incubation with primary antibody (anti-NCX1, 1:5000, ab177952, Abcam, Cambridge, UK; anti-Serca2a, 1:5000, A010-20, Badrilla, Leeds, UK and anti-GAPDH, 1:2000, cs5174, Cell Signaling Technologies, Danvers, MA, USA) in 0.5% MP/TBST overnight at 4 °C. Membranes were washed 3 times in TBST before incubation with secondary antibody (anti-rabbit HRP conjugated, 1:5000, NA934V, Cytvia, Malborough, MA, USA) for 1 h at RT and washed again 3 times in TBST.

For detection of chemiluminescence, membranes were incubated with Clarity Western ECL substrate (Bio-Rad Laboratories Inc., Hercules, USA) for 2–3 min and subsequently imaged in signal accumulation mode on the ChemiDoc^TM^ Touch Imaging System (Bio-Rad Laboratories Inc., Hercules, CA, USA). Densitometric quantification of bands was performed using ImageLab software (Bio-Rad Laboratories Inc., Hercules, CA, USA) using the “lanes and bands” tool with manual adjustments. Signal intensity of the protein of interest was normalized to corresponding intensity of the housekeeping protein (GAPDH).

### 2.8. RNA Extraction and Microarrays of Cardiac Tissue from Adult Dahl Salt-Sensitive Rats

RNA from left ventricular tissue was isolated using the microRNeasy^®^ Mini Kit (Qiagen, Hilden, Germany) according to the manufacturer’s protocol. RNA concentration was determined by NanoDrop^TM^ 2000 (Thermo Fisher Scientific, Waltham, MA, USA) before samples were shipped to ATLAS Biolabs (Berlin, Germany) for transcriptomic profiling using Affymetrix WT Expression Profiling Microarray Clariom D (Thermo Fisher Scientific, Waltham, MA, USA). For data analysis, CEL files were imported in transcriptome analysis console, Version 4.0 (Applied Biosystems, Waltham, MA, USA), and individual data points were exported. Values presented are log2 transformed mean signal intensities. Relevant transcript IDs and additional information are provided in Table S2.

### 2.9. Statistics

Statistical analysis was performed in GraphPad Prism 9 (San Diego, CA, USA). Data were tested for normality of distribution by Shapiro–Wilk test and D’Agostino–Pearson test.

In case of normal distribution and comparison of 2 groups, a *t*-test was applied. If the normality of distribution was violated, a Mann–Whitney test was conducted. For comparison of 3 groups, normal distribution was always violated; therefore, a Kruskal–Wallis test was conducted. In the case of comparison of trigger-induced changes to baseline levels of the same sample, a one-sample *t*-test was employed. For statistical analysis of grouped data (body weight and blood pressure curves), a mixed-effect analysis for overall effects and multiple unpaired *t*-tests for individual time points were employed. After correction for multiple testing where appropriate, a two-tailed *p* ≤ 0.05 was considered significant. The corresponding statistical tests for each data set are indicated in the figure legends.

## 3. Results

### 3.1. β-Adrenergic Signaling Is Essential for the Regulation of NCX Levels under Increased Workload

**Figure 1:**
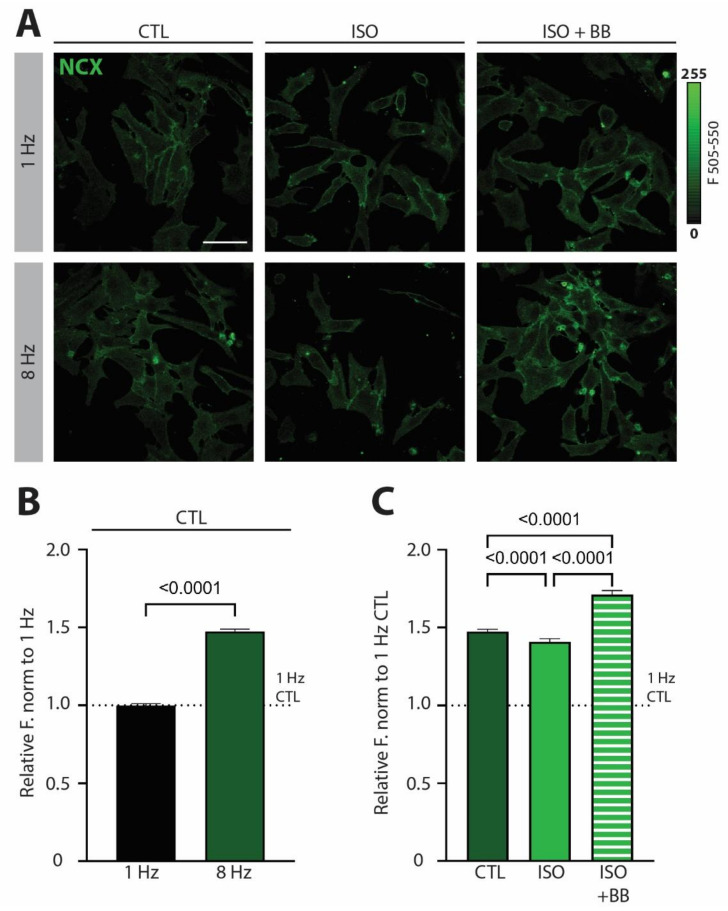
**β-adrenergic signaling prevents NCX upregulation under increased workload in *neonatal rat ventricular cardiomyocytes (NRVCMs)*.** (**A**) Representative fluorescent images of NRVCMs immunostained against NCX exposed to 3 h of low pacing (1 Hz) or tachycardic pacing (8 Hz) frequency under control conditions (CTL), β-adrenergic stimulation with isoprenaline (ISO) or β-adrenergic stimulation following 1 h preincubation with β-blocker propranolol (ISO + BB). Scale bar indicates 50 µm. (**B**) Mean fluorescent values of CTL under low pacing frequency (1 Hz) or tachycardia (8 Hz) normalized to the 1Hz condition. (**C**) Mean fluorescent values of CTL, ISO and ISO + BB at 8 Hz normalized to the CTL at 1 Hz. Data are shown as mean ± SEM (*n* = 760–1171 cells from *N* = 4 isolations). Indicated *p*-values were calculated using (**B**) Mann–Whitney test comparing 8 Hz to 1 Hz under CTL conditions or (**C**) Kruskal–Wallis test comparing normalized CTL, ISO or ISO + BB levels at 8 Hz.

Our previous work demonstrated that NCX is involved in tachycardia-dependent gene reprogramming in NRVCMs [5]. To test whether NCX expression levels are changed by high pacing frequency, we first exposed NRVCMs to a low (1 Hz) or high (8 Hz) frequency of electrical stimulation. We observed a significant upregulation of NCX expression on a protein level in NRVCMs exposed to tachycardic pacing (Figure 1A,B). This effect was partially abolished in the presence of β-adrenergic stimulation with isoprenaline (ISO; Figure 1A,C). In contrast, preincubation of cells with β-blocker propranolol (ISO + BB) completely reversed the ISO-induced effects. Even more so, in ISO + BB treated cells, NCX protein expression was increased to levels higher than in the absence of ISO (Figure 1C), suggesting that tachypacing is accompanied by limited autocrine or paracrine activation of β-adrenergic signaling.

These experiments suggest that β-adrenergic signaling plays an important role in the regulation of NCX expression under acute tachycardic stress by preventing its overt upregulation. This raises interesting questions as to whether altered NCX expression due to tachycardia can induce changes in Ca^2+^-mediated transcriptional regulation and whether β-adrenergic signaling downregulation and/or desensitization can cause NCX upregulation and downstream gene reprograming in conditions of cardiac pathologies.

### 3.2. β-Adrenergic Signaling Changes the Subcellular Pattern of CaMKII Overactivation

**Figure 2:**
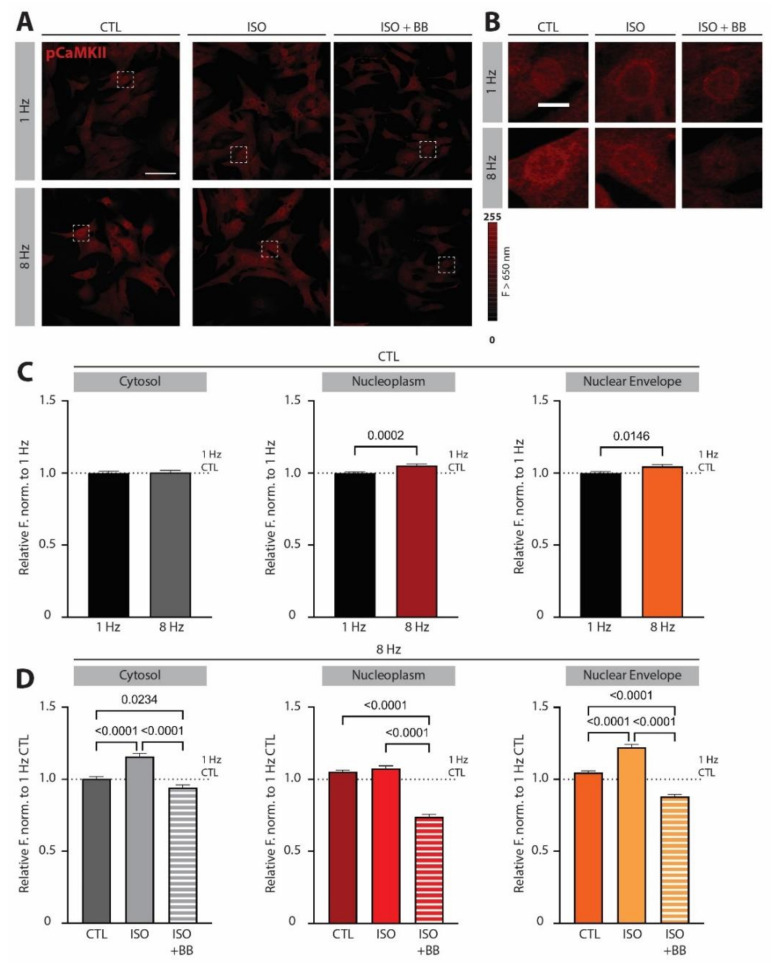
**β-adrenergic signaling potentiates CaMKII activation on subcellular Ca^2+^ stores in *neonatal rat ventricular cardiomyocytes (NRVCMs)* under conditions of tachypacing.** (**A**) Representative fluorescent images of *NRVCMs* immunostained against phospho-CaMKII (pCaMKII) exposed to 3 h of either low pacing (1 Hz) or tachycardic pacing (8 Hz) frequency under control conditions (CTL), β-adrenergic stimulation with isoprenaline (ISO) or β-adrenergic stimulation following 1 h preincubation with β-blocker propranolol (ISO + BB). Scale bar indicates 50 µm. (**B**) Magnified nuclei of indicated areas in (**A**). Scale bar indicates 10 µm. (**C**) Mean fluorescent values of CTL under low pacing frequency (1 Hz) or tachycardia (8 Hz) in cytoplasm, in nucleoplasm and on the nuclear envelope normalized to the 1 Hz CTL signal of the respective compartment. (**D**) Mean fluorescent values of CTL, ISO and ISO + BB conditions at 8 Hz normalized to the CTL at 1 Hz in cytoplasm, in nucleoplasm and on the nuclear envelope. Data are shown as mean ± SEM (*n* = 308–415 cells from *N* = 4 isolations). Indicated *p*-values were calculated using (**C**) Mann–Whitney test comparing 8 Hz to 1 Hz in the respective compartments or (**D**) Kruskal–Wallis test comparing normalized CTL, ISO and ISO + BB levels at 8 Hz in the respective compartments.

Tachycardic pacing has been shown to elevate CaMKII activation, which occurs downstream of NCX [5]. We next investigated whether β-adrenergic stimulation, which we showed to be effective in preventing NCX overexpression, is also capable of controlling CaMKII overactivation in conditions of tachycardic pacing. Interestingly, we found that tachycardic pacing in the absence of β-adrenergic stimulation led to selective increase in phospho-CaMKII (pCaMKII) in the nucleoplasm and on the nuclear envelope, while it had no effects on pCaMKII in the cytoplasm of NRVCMs (Figure 2A–C). Application of ISO selectively potentiated CaMKII activation in the cytoplasm and especially on the nuclear envelope under tachypacing conditions (Figure 2A,B,D). This subcellular redistribution of activated CaMKII may have important functional consequences, as we previously demonstrated that CaMKII activation on the nuclear envelope effectively protects the nucleus from Ca^2+^ overload and promotes an adaptive transcriptional response, while activation of the CaMKII axis in the nucleus contributes to eccentric hypertrophy and the progression of maladaptive cardiac remodeling [9].

Treatment with ISO + BB led to a striking reduction in pCaMKII activation to levels below those observed at baseline pacing frequency under CTL conditions at 1 Hz in all subcellular compartments, likely suggesting a limited but significant autocrine or paracrine activation of β-adrenergic signaling in electrically stimulated cells already at 1 Hz (Figure 2A,B,D). However, we cannot exclude the possibility that off-target effects of BB additionally influence CaMKII phosphorylation [14].

### 3.3. β-Adrenergic Signaling Prevents *IL6R* Signaling Overactivation upon Increased Workload

**Figure 3:**
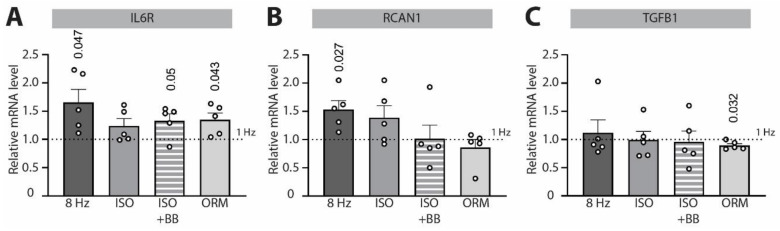
**Effects of tachycardic pacing and β-adrenergic stimulation/inhibition on the expression of hypertrophy- and cardiac-remodeling-relevant genes in *neonatal rat ventricular cardiomyocytes (NRVCMs)*.** Relative expression of (**A**) interleukin-6 receptor (*IL6R*), (**B**) regulator of calcineurin 1 (*RCAN1*) and (**C**) transforming growth factor β 1 (*TGFB1*) exposed to 3 h of tachycardia (8 Hz) under control conditions or combined with β-adrenergic stimulation with isoprenaline (ISO), β-adrenergic stimulation following 1 h preincubation with β-blocker propranolol (ISO + BB) or treatment with NCX inhibitor ORM-10962 (ORM). All 8 Hz conditions were normalized to the respective low pacing frequency (1 Hz) condition (using the 2^−ΔΔCt^ method). Data are shown as mean ± SEM (*N* = 5 isolations). Indicated *p*-values were calculated using a one-sample *t*-test.

To test the potential transcriptional downstream effects of the observed NCX-pCaMKII changes induced by tachypacing and β-adrenergic signaling, we screened hypertrophy- and cardiac-remodeling-relevant genes for changes in mRNA expression levels under the same experimental conditions we employed for immunocytochemistry experiments (Figure 3). Here, we observed that tachypacing alone enhanced *IL6R* gene transcription, while this effect was abolished by ISO treatment (Figure 3A). To test whether prevention of NCX upregulation or selective enhancement of CaMKII activity on the sarcoplasmic reticulum or on the nuclear envelope are predominantly responsible for the ISO-mediated effect, we treated *NRVCMs* with either ISO + BB or selective NCX inhibitor ORM-10962. Neither of the two treatments was effective in preventing *IL6R* overactivation (Figure 3A), suggesting that simultaneous prevention of NCX overexpression and CaMKII activation on subcellular Ca^2+^ stores are needed to abolish overactivated *IL6R*-signaling under increased workload. This is in alignment with our previous findings that in the absence of ISO, NCX inhibition attenuates CaMKII phosphorylation upon tachypacing [5], while CaMKII inhibition leads to a significant increase in *IL6R* expression under the same experimental conditions [9]. Of note, the reversal of ISO-induced downregulation of *IL6R* by BB and ORM-10962 appears partial, and it might suggest the existence of alternative pathways involved in *IL6R* signaling regulation. *RCAN1* was also significantly upregulated by tachycardic pacing; however, its expression could only be abolished in conditions where CaMKII activation was inhibited (upon ISO + BB or ORM-10962 treatment; Figure 3B). In contrast, we detected no effects of ISO on the mRNA expression of TGFβ1 (Figure 3C), while ORM-10962 exhibited a tachycardia-independent effect by downregulating its expression. This suggests the specific effect of β-adrenergic signaling on the regulation of *IL6R* signaling under increased cardiac workload.

### 3.4. Blunted β-Adrenergic Signaling Due to Hypertensive Cardiac Remodeling Is Associated with Increased NCX Expression and IL6R Signaling

To test the pathophysiological relevance of the newly identified role of β-adrenergic stimulation in preventing increased NCX expression and *IL6R* signaling, we further investigated the potential impairment of this pathway in hypertension-induced cardiac remodeling. Although the etiology of cardiac remodeling in high-salt diet fed *Dahl salt-sensitive rats* is different than tachypacing-induced changes in *NRVCMs*, these rats are characterized by blunted β-adrenergic response [15], which may fail to maintain proper regulation of NCX and *IL6R* expression in their chronically pressure-overloaded hearts. In vivo characterization of *Dahl salt-sensitive rats* fed high-salt diet (8% NaCl) for 10 weeks confirmed significantly elevated diastolic and systolic blood pressure compared to the low-salt diet (0.3% NaCl)-fed control group (Figure S1A–C). Echocardiographic assessment showed that increased BP in high-salt diet fed rats was not associated with the reduction in ejection fraction (Figure S1D) or body weight loss (Figure S1E). However, those rats showed clear signs of diastolic dysfunction, with significantly elevated E/e′ ratios (Figure S1F) and left ventricular (Figure S1G), heart (Figure S1H) and cellular (cell length and width Figure S1I,J, respectively) hypertrophy.

**Figure 4:**
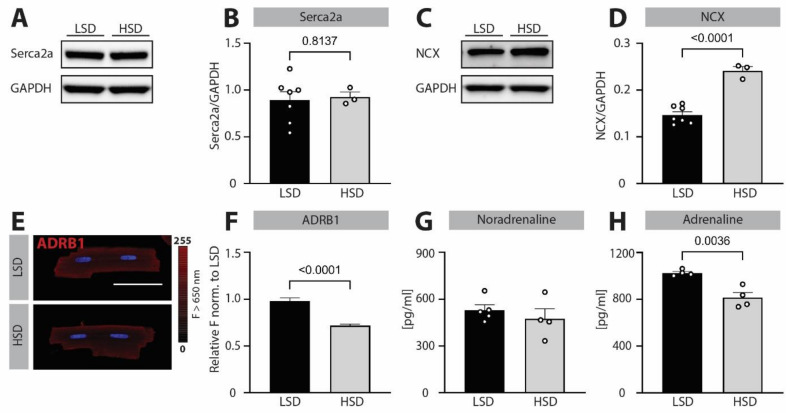
**Hypertension is associated with blunted β-adrenergic signaling and increased NCX expression in *Dahl salt-sensitive rats*.** (**A**) Representative bands of immunoblotted Serca2a and housekeeping protein GAPDH in cardiac tissue of *Dahl salt-sensitive rats* kept on low-salt diet (LSD) or high-salt diet (HSD) for 10 weeks. (**B**) Corresponding quantification of Serca2a expression, normalized to GAPDH. (**C**) Representative bands of immunoblotted NCX and housekeeping protein GAPDH in cardiac tissue of *Dahl salt-sensitive rats* kept on LSD or HSD for 10 weeks. (**D**) Corresponding mean values of NCX expression, normalized to GAPDH. (**E**) Representative fluorescent images of isolated cardiomyocytes immunostained against β-adrenergic receptor 1 (ADRB1). Scale bar indicates 50 µm. (**F**) Mean fluorescent values for ADRB1 normalized to LSD. Mean values for (**G**) noradrenaline and (**H**) adrenaline plasma levels in *Dahl salt-sensitive rats* kept on LSD or HSD for 10 weeks. Data are shown as mean ± SEM (*N* = 7/3 rats for LSD/HSD for B and D, *N* = 2/2 rats and *n* = 76/79 cells for LSD/HSD and *N* = 4–5/4 rats for LSD/HSD). Indicated *p*-values were calculated using unpaired *t*-test (**B**,**D**,**G**,**H**) or Mann–Whitney test (**F**) comparing HSD to LSD.

**Figure 5:**
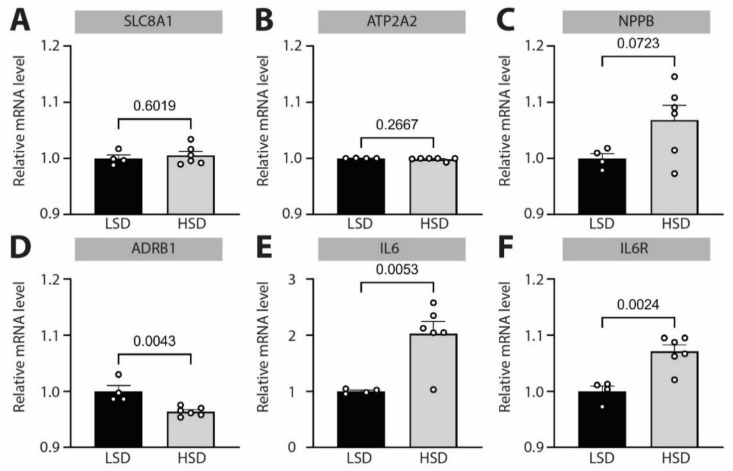
**Effects of hypertension on the expression of disease-relevant mRNAs in *Dahl salt-sensitive rats.****Dahl salt-sensitive rats* were kept on low-salt diet (LSD) or high-salt diet (HSD) for 10 weeks to induce hypertension. mRNA levels of (**A**) *SLC8A1* (gene encoding for NCX), (**B**) *ATP2A2* (gene encoding for Serca2a), (**C**) brain natriuretic peptide (NPPB), (**D**) β-adrenergic receptor 1 (*ADRB1*), (**E**) interleukin-6 (*IL6*) and (**F**) interleukin-6 receptor (*IL6R*) were quantified by microarray analysis and normalized to the mean expression level of LSD. Data are shown as mean ± SEM (*N* = 5/6 rats for LSD/HSD). Indicated *p*-values were calculated using unpaired *t*-test (except for B, where a Mann–Whitney test was applied).

In line with these results, in vitro phenotyping showed no downregulation of Serca2a expression at a protein or mRNA level (Figure 4A,B and Figure 5B), which would be a typical feature of heart failure with reduced ejection fraction [16,17]. In contrast, we observed strongly increased expression of NCX in high-salt diet fed rats (Figure 4C,D) despite the mRNA levels remaining unchanged (Figure 5A), underlining the predominant pathophysiological importance of NCX dysregulation in diastolic-dysfunction-related cardiac disease. Furthermore, we found significant downregulation of β-adrenergic receptor 1 expression at both a protein (Figure 4E,F) and mRNA level (Figure 5D), with no change in noradrenaline and decrease in adrenaline plasma levels (Figure 4G,H), pointing to an overall blunted β-adrenergic signaling in hypertensive animals.

Finally, to investigate if observed dysregulation of NCX expression and β-adrenergic receptor 1 signaling affects similar downstream pathways, as seen in experiments with electrically stimulated *NRVCMs*, we performed high-throughput screening of mRNA expression levels by microarray analysis (Figure 5 and Table S2). Analyses of hypertrophy-related genes supported the echocardiographic, gravimetric and cellular analyses of cardiac hypertrophy with increased cardiac hypertrophic marker NPPB (Figure 5C). Strikingly, we identified the *IL6R* pathway as one of the most affected signaling cascades in hypertension-induced cardiac remodeling, which is characterized by simultaneous β-adrenergic receptor 1 downregulation and NCX upregulation. Namely, there was a two-fold upregulation of *IL6* (Figure 5E, note different scale of *y*-axis), and significant upregulation of the *IL6R* (Figure 5F), underscoring the importance of intact β-adrenergic signaling for preventing *IL6R* overactivation under increased workload, which presents itself as high-frequency beating or increased afterload due to hypertension.

## 4. Discussion

The results of the present study indicate that acute β-adrenergic receptor stimulation prevents overactivation of *IL6R*-signaling in the heart under physiological stress, thereby preventing transcriptional remodeling and maladaptive response to short-term increases in cardiac workload. Mechanistically, two features of β-adrenergic signaling are associated with this effect: (1) prevention of NCX overexpression and (2) potentiation of CaMKII signaling, specifically on intracellular Ca^2+^ stores, the sarcoplasmic reticulum and nuclear envelope. In addition, conditions of chronic β-adrenergic signaling depression are characterized by NCX upregulation and are followed by a dramatic increase in *IL6R* signaling. These data could help the refinement of existing cardioprotective strategies, especially for patients commencing β-blocker therapy and/or those with advanced hypertension.

Cardiomyocytes can directly release *IL6* in response to injury and cardiac stress. The duration of *IL6* production and signaling by cardiomyocytes determines whether this signaling is protective and capable of limiting cellular damage or detrimental, with the latter leading to chronically depressed cardiac function [18]. In response to injury, *IL6* acutely diminishes cardiomyocyte contractility, activates anti-apoptotic signaling and preserves the viability of the myocardium [19,20]. However, both animal and human studies have showed that if *IL6* signaling remains elevated after the immediate need to preserve the insulted tissue has resolved, reduced contractility of the myocardium induces activation of hypertrophic gene signaling, which can eventually lead to heart failure [21,22]. For example, IL6-family signaling was shown to decrease the basal contractility of cardiomyocytes and their β-adrenergic responsiveness, ultimately leading to decreased function [23,24], as well as induction of gene expression that is essential for the development of pathological hypertrophy via activating CaMKII and STAT3 pathways [25,26]. In contrast, inhibition of *IL6* signaling by disrupting IL6/gp130 interaction reduced pressure-overload-induced cardiac inflammation, remodeling and dysfunction [27]. Thus, signaling downstream of *IL6* without acute insult to cardiomyocytes can be a detrimental feature that facilitates changes in the myocardium, leading from a physiological to a pathological state.

Although the individual roles of excessive β-adrenergic and *IL6* stimulation in the pathogenesis of cardiomyocyte hypertrophy are well documented, their crosstalk in cardiomyocytes remains controversial. Szabo-Fresnais et al. [28] observed an enhancement of *IL6* production when ISO was applied to adult ventricular myocytes. Similarly, chronic β-adrenergic stimulation induced myocardial proinflammatory cytokine—including IL6—expression, according to Murray et al. [29]. In our study, however, acute β-adrenergic stimulation attenuated a tachypacing-induced increase in *IL6* expression (Figure 3). These seemingly opposing results may be a consequence of the absence (in the study by Szabo-Fresnais et al.) or presence (in our study) of high-pacing-frequency electrical stimulation of cardiomyocytes, which may be critical for qualitative features of β-adrenergic stimulation-driven effects. Murray et al. applied chronic ISO stress via miniosmotic pumps implanted in rats for 7 days; however, they did not assess the potential downregulation or desensitization of β-adrenergic signaling that typically occurs in cardiomyocytes upon upregulation of the β-adrenergic tone. Therefore, it is not possible to assess whether *IL6* overexpression occurs as a result of increased or blunted β-adrenergic response in their study [29]. In agreement with our data, while ISO was capable of inducing an increase in *IL6* levels in cardiac fibroblasts, it did not cause such response in cultured cardiomyocytes [30]. Furthermore, it has been recently shown that ISO inhibits transcription of cardiac cytokine genes—including IL6—induced by reactive oxygen intermediates [31], in a similar fashion to its prevention of *IL6* upregulation due to tachycardia. In addition, as effects of β-adrenergic signaling appear to be highly time-dependent, one has to pay close attention to differential outcomes upon acute vs. chronic β-adrenergic stimulation.

An intriguing aspect of our previous results is that CaMKII inhibition under conditions of tachypacing strongly promoted cardiomyocyte hypertrophy and the expression of NPPB and *IL6* [9]. We therefore assessed the effect of β-adrenergic stimulation on CaMKII activation upon tachypacing and discovered that—unlike tachycardia alone, which mostly activates CaMKII in the nucleoplasm—ISO treatment potentiates CaMKII activation specifically on the intracellular Ca^2+^ stores, while it has no effect on nucleoplasmic CaMKII activation (Figure 2). This specific spatial activation pattern of CaMKII may be an adaptive mechanism and may keep *IL6* expression low upon acute increase in workload, likely due to the enhanced sarcoplasmic reticulum and nuclear envelope Ca^2+^ uptake that prevents nuclear Ca^2+^ overload [9]. In line with this idea, a study by Baier et al. [32] showed that excessive CaMKII inactivation in AC3-I (autocamtide-3-derived inhibitory peptide) transgenic mice leads to diminished cardiac function and premature death in the first days after inducing pressure overload, highlighting CaMKII activation as an essential element of cardiac stress physiology.

When we initially studied the effects of tachycardia on cardiomyocyte gene reprograming, we found that high pacing frequency alone increases CaMKII activation without an evident accumulation of CaMKII on the nuclear envelope, while nucleoplasmic activation was evident [5]. Using pharmacological inhibitors of various Ca^2+^-regulating proteins, we could also show that this effect occurs downstream of NCX. Although acute application of β-adrenergic stimulating agents has no immediate effect on NCX current in intact rabbit, guinea pig, mouse, and rat ventricular myocytes at low pacing frequencies [33,34], 72 h of in vivo β-adrenergic treatment induced an increase in NCX expression [35]. Strikingly, no cellular or in vivo data are available for the effects of β-adrenergic stimulation in tachycardic conditions. In our experiments, short-term ISO treatment partially prevented tachypacing-induced NCX upregulation, while the addition of ISO + BB completely reversed the effect, thereby highlighting the importance of including (tachy)pacing in the experimental protocol when interpreting data on the effect of β-adrenergic stimulation on physiological stress. On the other hand, numerous studies involving in vivo tachypacing procedure are available, and they readily support our finding that intact β-adrenergic receptor signaling is needed to maintain proper NCX activity under cardiac stress. For example, Briston et al. showed that 5 weeks of in vivo right ventricular tachypacing leads to dysfunctional β-adrenergic receptor signaling with reduced cardiomyocytes responsiveness to β-adrenergic receptor agonists, which is paralleled by greater NCX activity and protein expression [36]. Although beyond the scope of the current study, measurements of NCX current and activity (e.g., by an altered relaxation of the caffeine-induced Ca^2+^ transient) are needed to address the acute functional consequences of rapid-pacing-induced NCX upregulation and the potency of β-adrenergic stimulation in preventing this effect under short-term physiological stress.

**Figure 6:**
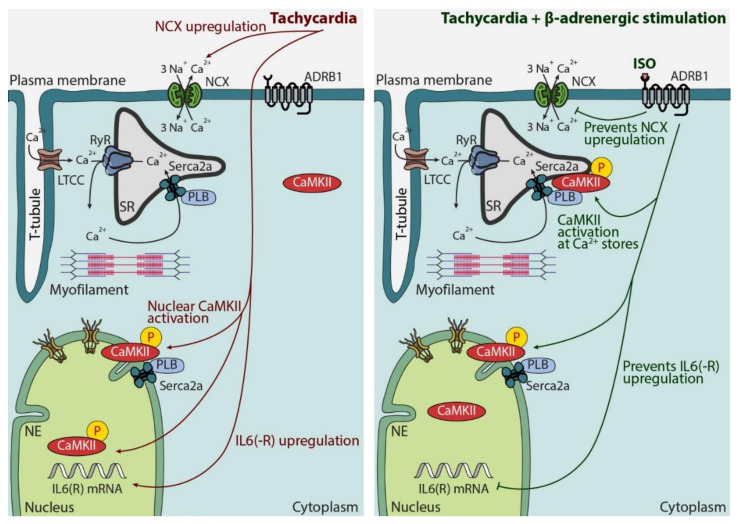
**Graphical representation of effects of tachycardia alone, which are abolished by tachycardia combined withβ-adrenergic stimulation, on expression of NCX, subcellular CaMKII activation profile and *IL6(R)* in cardiomyocytes.** Abbreviations: ADRB1—β-adrenergic receptor 1, CaMKII—calcium/calmodulin-dependent kinase II, *IL6(R)*—interleukin 6 (receptor), ISO—isoprenaline, LTCC—L-type calcium channel, NCX—sodium/calcium exchanger, NE—nuclear envelope, PLB—phospholamban, RyR—ryanodine receptor, Serca2a—sarcoplasmic reticulum calcium ATPase 2a, SR—sarcoplasmic reticulum.

Our working hypothesis, based on cellular experiments and qPCR data, is the following (Figure 6): In a healthy cardiomyocyte, tachypacing induces nuclear CaMKII activation and NCX and *IL6R* upregulation. Unlike *IL6R* upregulation, it is less likely that altered transcription is the main cause of NCX upregulation—as its mRNA levels remained stable under our experimental conditions ([5] and Figure 5A)—but rather increased trafficking and/or decreased degradation. In the presence of β-adrenergic stimulation, however, CaMKII activation is selectively potentiated on intracellular Ca^2+^ stores, while NCX and *IL6* upregulation are prevented. Both downstream effects of β-adrenergic stimulation (i.e., CaMKII activation on the nuclear envelope and inhibition of excessive NCX upregulation) are needed to prevent IL6-signaling overactivation upon short-term increase in workload (Figure 1, Figure 2 and Figure 3).

Short-term β-adrenergic receptor stimulation appears essential in preventing inflammatory response during acute stress, which may appear counterintuitive due to the well-documented advantages of BB in treating heart failure. However, in chronic settings, BB treatment prevents the downregulation and/or desensitization of β-adrenergic receptors [2] and thereby maintains—at least partial—functionality of the receptors. This is likely to maintain cardiomyocytes’ responsiveness to catecholamines (and thus exercise tolerance), but it can also contribute to controlling their inflammatory signaling.

Although chronic β-adrenergic tone is readily implicated in the development of cardiac pathologies, while acute effects are known for instantaneous regulation of cellular Ca^2+^ cycling and contractility, little is known about the role of β-adrenergic signaling in preventing gene reprograming under short-term physiological stress. We previously showed that ISO keeps histone deacetylase 4 (HDAC4) localized in the nucleus in the very early response to physiological challenge [37]. Maccari et al. showed that β-adrenergic signaling also prevents the upregulation of β-myosin heavy chain (β-MHC), and both effects are associated with preventing cardiac dysfunction [37,38]. Our present data identify a novel β-adrenergic-signaling-dependent mechanism for controlling transcriptional reprograming during short-term increase in workload demands of the heart. Functional integrity of this cellular mechanism is a prerequisite for keeping *IL6R* signaling low and preventing inflammatory response during physiological stress.

To tackle the question of clinical relevance of this newly discovered signaling cascade downstream to β-adrenergic receptor, we studied *Dahl salt-sensitive rats* fed high-salt diet for 10 weeks. Upon being treated with our feeding protocol (Figure S1A), these rats developed severe hypertension, diastolic dysfunction and cardiac hypertrophy on an organ and cardiomyocyte level (Figure S1), and most importantly for this study, they showed depressed β-adrenergic signaling (Figure 4), which is typically observed in cardiac pathologies in the clinical setting [39,40]. We showed that reduced β-adrenergic signaling was associated with strong NCX upregulation and that enhancement of *IL6R*-signaling was one of the most affected pathways in microarray analyses (Figure 5). According to our cellular data, such loss in β-adrenergic responsiveness would upregulate NCX under tachycardic pacing stress, an effect likely potentiated by the mechanical stress to individual myocytes caused by hypertension [5], thus together leading to *IL6R* upregulation. From our data, we cannot completely rule out the involvement of other pathways with downstream effects on *IL6R* in two etiologically distinct models used in the study. However, observed changes in *NRVCMs* are completely recapitulated in the adult *Dahl salt-sensitive rats*, arguing for at least partial activation of the same signaling pathway in both models and the more universal role of β-adrenergic receptor signaling in preventing inflammatory overactivation. In line with this, adverse diastolic phenotype—similar to that observed in our hypertensive rat model—was previously associated with enhanced *IL6* signaling. For example, the BIOSTAT-CHF (A Systems Biology Study to Tailored Treatment in Chronic Heart Failure) trial data demonstrated that suffering from heart failure with preserved ejection fraction (HFpEF) was a strong independent predictor of increased *IL6* levels [41], while the PREVEND (Prevention of Renal and Vascular End-Stage Disease) study found *IL6* levels significantly associated with the development of HFpEF but not heart failure with reduced ejection fraction (HFrEF) [42]. Our data support the need for further research to investigate whether *IL6* might be a potential novel treatment target to halt or even prevent HFpEF.

One important point of consideration is the origin of *IL6* and *IL6R* in myocardial tissue of hypertensive *Dahl salt-sensitive rats* detected in microarray analyses, as *IL6* can be secreted by both human cardiomyocytes and cardiac fibroblasts [43]. A recent study by Kumar et al., however, showed that *IL6* expression in cardiac fibroblasts cannot be stimulated by transverse aortic constriction or exposure to pro-hypertrophic factors, including phenylephrine, angiotensin II, transforming growth factor-β, and hypoxia. In contrast, conditioned medium from cardiomyocytes with increased *IL6* production due to hypoxia-induced mitogenic factor (HIMF) can increase *IL6* production in fibroblasts and their proliferation, migration, and myofibroblast differentiation [26]. These data support that *IL6* signaling in cardiac fibrosis is activated via a cardiomyocyte-to-fibroblast paracrine effect, with *IL6* signaling in cardiomyocytes playing a central role in cardiomyocyte hypertrophy.

In summary, we have demonstrated that β-adrenergic stimulation has a direct effect on NCX protein expression and downstream CaMKII subcellular activation/localization upon increased workload of the heart, which prevents augmentation of *IL6R* signaling. We propose a previously unidentified protective role of short-term ISO stimulation in inhibiting *IL6R* activation and NCX dysregulation. A better understanding of these processes may contribute to refinement of existing therapeutic options for patients with compromised β-adrenergic signaling.

## Data Availability

The data supporting findings of this study are available from the corresponding authors upon reasonable request. Materials and methods and expanded data sections not included in the text body are described in detail in the Supplementary Materials.

## Supplementary Materials

The following supporting information can be found at https://www.mdpi.com/article/10.3390/biomedicines10071648/s1: Figure S1: *Dahl salt-sensitive rats* develop hypertension, diastolic dysfunction and cardiac hypertrophy upon high-salt diet (HSD, 8% NaCl) feeding compared to low-salt diet (LSD, 0.3% NaCl); Table S1: List of TaqMan^TM^ Assays; Table S2: Additional microarray information for RNA analysis of *Dahl salt-sensitive rats* on either high-salt (HSD) or low-salt diet (LSD) for 10 weeks.
