# Supplementary material for: β-Adrenergic Receptor Stimulation Maintains NCX-CaMKII Axis and Prevents Overactivation of IL6R-Signaling in Cardiomyocytes upon Increased Workload
Source: Biomedicines. 2022 Jul 8;10(7):1648. doi: 10.3390/biomedicines10071648 (PMC9313457; doi:10.3390/biomedicines10071648)
Supplement: Supplementary file 1 [file biomedicines-10-01648-s001.zip › biomedicines-1764619-supplementary.pdf]

biomedicines

MDPI

β-adrenergic receptor stimulation maintains NCX-CaMKII axis and prevents overactivation of IL6R-signaling in cardiomyocytes upon increased workload

Supplementary materials

Information on the diet plan and in vivo and hypertrophy phenotypisation of hypertensive rats is shown in Figure S1.

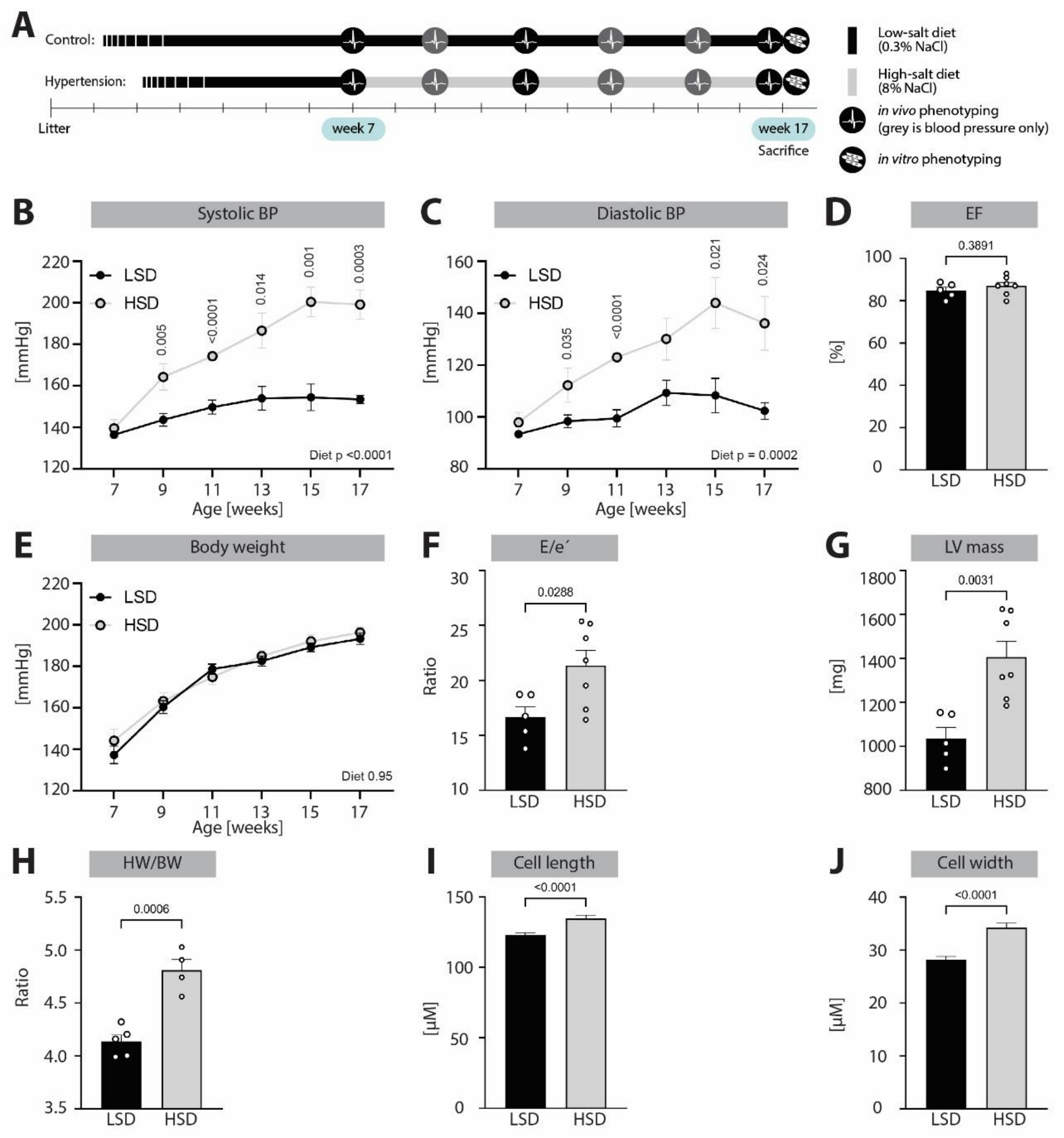

Figure S1. *Dahl salt-sensitive rats* develop hypertension, diastolic dysfunction and cardiac hypertrophy upon high-salt diet (HSD, 8% NaCl) feeding compared to low-salt diet (LSD, 0.3% NaCl). (**A**) Schematic overview of feeding and experimental plan of *Dahl salt-sensitive rats*, which were purchased at 4–5 weeks of age and fed either a LSD or HSD diet starting at the age of 7 weeks for 10 weeks thereafter. Blood pressure measurements were carried out biweekly, and echocardiographic phenotyping was performed at baseline (7 weeks) and presacrifice (17 weeks). (**B**) Systolic and (**C**) diastolic blood, (**D**) ejection fraction (EF), (**E**) body weight curve, (**F**) ratio of peak early Doppler transmitral flow velocity [E] to myocardial tissue Doppler velocity [e´], (**G**) left ventricular (LV) mass, (**H**) ratio of heart (HW) to body weight (BW), (**I**) cell length and (**J**) cell width. Data are

presented as mean ± SEM (*N* = 5–11/4-7 rats for LSD/HSD, and for I and J, *n* = 203/124 cells for LSD/HSD). Indicated *p*-values were calculated by unpaired *t*-test (**D**,**F–I**), Mann–Whitney test (**J**), multiple unpaired t-tests (vertical *p*-values in **B** and **C**) or mixed-effect analysis (**B**,**C** and **E** bottom left diet effect).

Relevant information on the employed TaqMan™ Gene Expression Assays is provided in Table S1. Additional information on transcript IDs and RNA levels (including fold change) of microarrays performed on RNA isolated from *Dahl salt-sensitive rats* is provided in Table S2.

Table S1. List of TaqMan™ Assays (Thermo Fisher Scientific, Waltham, MA, USA) used for qPCR in *NRVCMs*.

Target Gene Name	TaqMan™ Gene Expression Assay ID	LOT
Regulator of Calcineurin 1 (*RCAN1*)	Rn00596606_m1	1733914
Interleukin-6 Receptor (*IL6R*)	Rn01495381_m1	1975678
Transforming Growth Factor beta 1 (*TGFB1*)	Rn00572010_m1	1984434
GAPDH	Rn01775763_g1	1992407

Table S2. Additional microarray information for RNA analysis of *Dahl salt-sensitive* rats on either high-salt (HSD) or low-salt diet (LSD) for 10 weeks.

ID	HSD Avg (log2)	LSD Avg (log2)	Fold Change	*p*-Value	FDR *p*-Value	Gene Symbol
TC0600001773.rn.1	13.06	13	1.04	0.5803	0.8688	Slc8a1
TC1200000643.rn.1	19.03	19.03	−1	0.5656	0.8627	Atp2a2
TC0500001897.rn.1	15.3	14.21	2.13	0.0144	0.1954	Nppb
TC0100003865.rn.1	8.83	9.07	−1.18	0.0597	0.3863	Adrb1
TC0400000061.rn.1	8.62	3.99	24.8	7.44×10^{-5}	0.0081	Il6
TC0200004086.rn.1	6.95	6.51	1.36	0.0001	0.0115	Il6r
